# The UCLA Cosmochemistry Database

**DOI:** 10.1038/s41597-023-02807-7

**Published:** 2023-12-07

**Authors:** Bidong Zhang, Kerstin A. Lehnert, Alan E. Rubin, Kevin D. McKeegan, Paul H. Warren, Jennifer L. Mays, Lucia R. Profeta, Annika K. Johansson, Peng Ni, Edward D. Young, Frank T. Kyte, Ming-Chang Liu, Emilie T. Dunham, Haolan Tang, Peng Ji, Juan D. Figueroa-Salazar

**Affiliations:** 1 Department of Earth, Planetary, and Space Sciences, University of California, Los Angeles, CA 90095-1567 USA; 2 Lamont-Doherty Earth Observatory, Columbia University, Palisades, NY 10964 USA; 3 Maine Mineral & Gem Museum, 99 Main Street, P.O. Box 500, Bethel, ME 04217 USA; 4 Lawrence Livermore National Laboratory, Livermore, CA 94550 USA; 5 School of Earth and Space Sciences, University of Science and Technology of China, Hefei, Anhui 230026 China

**Keywords:** Geochemistry, Early solar system

## Abstract

The UCLA Cosmochemistry Database was initiated as part of a data-rescue and -storage project aimed at archiving a variety of cosmochemical data acquired at University of California, Los Angeles (UCLA). The data collection includes elemental compositions of extraterrestrial materials analyzed by UCLA cosmochemists over the last five decades. The analytical techniques include atomic absorption spectrometry (AAS) and neutron activation analysis (NAA) at UCLA. The data collection is stored on the Astromaterials Data System (Astromat). We provide both interactive tables and downloadable datasheets for users to access all data. The UCLA Cosmochemistry Database archives cosmochemical data that are essential tools for increasing our understanding of the nature and origin of extraterrestrial materials. Future studies can reference the data collection in the examination, analysis, and classification of newly acquired extraterrestrial samples.

## Background & Summary

The current collections of extraterrestrial materials include meteorites and mission-returned samples from the Moon, asteroids (e.g., Ryugu by Hayabusa 2), a cometary coma (Stardust), and the solar wind (Genesis). The chemical and isotopic compositions of extraterrestrial materials are essential aspects of our knowledge of the Solar System. The UCLA meteorite team has analyzed the elemental compositions of a wide range of extraterrestrial materials including stony, stony-iron and iron meteorites, and lunar samples since the 1960s, and has accumulated a large quantity of data.

These UCLA data have mostly been published in journal articles, but most of the published data were neither digitized nor stored in a single repository that would allow easy access and discovery by the meteoritical community. The UCLA Cosmochemistry Database is part of our endeavor to compile all the cosmochemical data collected by the UCLA meteorite team. The UCLA Cosmochemistry Database aims to provide a freely accessible, web-based platform that allows the meteoritical community and general public to browse and use these data. We also envisage the UCLA Cosmochemistry Database as an example of rescuing historical, undigitized geochemical data. The UCLA team works with the Astromaterials Data System (Astromat, https://www.astromat.org/) to build and operate the UCLA Cosmochemistry Database, and it is one of the data collections stored in Astromat. Both the UCLA and Astromat teams provide continuous input, maintenance, and improvement to the collection.

The UCLA Cosmochemistry Database so far includes downloadable datasheets (Microsoft® Excel® files). Fifty-four publications^1–54^ by the UCLA meteorite team have been digitized as datasheets, which are stored in the Astromat repository (AstroRepo). Tabular data from spreadsheets and metadata of files are incorporated into a relational database (AstroDB Synthesis), that can be searched and accessed via an interactive web interface and APIs. The compositional data were collected by instrumental neutron activation analysis (INAA), radiochemical neutron activation analysis (RNAA), and atomic absorption spectrometry (AAS).

## Methods

We digitized the tables and supplementary information that have NAA and AAS data, including mean values and replicates. Data digitization of the publications was undertaken by the “Data from Picture” feature in Office 365 Excel. PDF files of publications were first converted to high-resolution (1200 ppi) JPEG images. Data tables were imported to Excel and converted to Excel spreadsheets. Because the converted tables may not meet Astromat’s requirements of formatting and the data accuracy was compromised during the conversion processes, the converted data were reviewed cell-by-cell to ensure these are consistent with the data in the publications, and the tables were reformatted to comply with the Astromat data submission guidelines. All digitized data have the same digits and decimal numbers as in the original publications. Typos in the original tables were corrected.

## Data Records

All data^55–108^ are available at Astromat. DOI of each publication can be found in the reference list. All data are accessible as an independent Astromat collection named “UCLA Cosmochemistry Database” (https://www.astromat.org/collections/ucla-cosmochemistry-database/). In the collection, users may search and browse all data by applying filters. We currently compiled elemental compositions of iron meteorites, pallasites, mesosiderites, chondrites, and achondrites.

The dataset repository currently archives data from the publications that contain AAS, INAA, and/or RNAA procedures performed at UCLA. We have digitized tables containing elemental compositions (both mean and replicate data) in 54 journal articles^1–54^ (primarily papers on iron and stony-iron meteorites, and chondrites). Each of these publications is individually archived into an Astromaterials Data Archive template (a Microsoft Excel 97–2004 worksheet), and each of the archive templates is defined as a dataset. Every dataset has been assigned its own DOI so that it can be cited independently. For example, for the original journal article:

Wasson, J. T. (1990). Ungrouped iron meteorites in Antarctica: Origin of anomalously high abundance. *Science*, 249(4971), 900–902. 10.1126/science.249.4971.900.

its corresponding Astromat dataset is:

Wasson, J. T. and Zhang, B. (2021). Data from “Ungrouped iron meteorites in Antarctica: Origin of anomalously high abundance” by Wasson (1990), Version 1.0. *Interdisciplinary Earth Data Alliance (IEDA)*. 10.26022/IEDA/112116.

Each Excel spreadsheet has seven tabs: 1. Data source, 2. Notes on the data compilation, 3. Samples, 4. Analytical data, 5. Primary method metadata, 6. Method-specific metadata, and 7. Vocabularies. The “Data source” tab includes information about the title, abstract, and submitter of the dataset as well as publications related to the dataset. The “Notes on the data compilation” tab records additional information from the submitter. These notes include clarification on the nomenclature of meteorites and technical details that are not shown in other tabs. The “Samples” tab shows the original names of meteorites used in the publication, and the “JSC Sample ID” and “Lithology” columns show the official full names and classification, respectively, of meteorites as recorded in the Meteoritical Bulletin Database. The “Data Source” column is the corresponding table title and number of samples. The “Analytical data” tab has columns “Sample Names” (original names of meteorites in the publication”, “Description” (table number of data), “Calculated average” (if the data are calculated from multiple replicates), and elemental compositions of samples. The tab “Primary method metadata” has additional information on the analytical data, including analytical techniques, laboratory, and analyst. The “Method-specific metadata” tab is usually vacant in our currently datasets. The last tab, “Vocabularies”, contains the suggested vocabularies for AstroRepo data submissions: parameter, technique, and unit.

Once the data and metadata in the datasheets are ingested into the AstroDB synthesis, users may search the synthesis database and apply filters such as mission number (specifically for Apollo samples), taxon (meteorite classes), analysis type (whole rock, phases), and variable (analytes) to create customized datasets with desired data for download.

## Technical Validation

All elemental analyses performed by the UCLA meteorite team are validated by respective reference materials, and all data are published in peer-reviewed journal articles. For NAA data, most samples were analyzed at least twice to ensure the reproducibility of the data. The analytical details are discussed in each of the digitized publications.

## Usage Notes

For the NAA data, iron meteorites were reanalyzed as the technique improved. Old data were constantly recalibrated, and their weighted-mean calculations are from replicates analyzed at different times. Datasheets containing the recommended mean value and replicates for each iron meteorite are uploaded to the repository. Users may find that the compositions of iron meteorites from the previous UCLA publications are different from the recommended compositions in the UCLA Cosmochemistry Database. The data from publications, especially those before 1999, are not necessarily the currently recommended values. We have made our recommendations on the composition of each iron meteorite based on our most recent analyses and calculations. Users are advised to use these recommended compositions for their iron-meteorite studies.

Note that the Astromat datasheets record the original meteorite names and classification information in the publications, and the classification of iron meteorites may have later been modified in the Meteoritical Bulletin Database. We encourage users to refer to the Meteoritical Bulletin Database for up-to-date classifications of individual iron meteorites. We encourage users to examine new classification, pairing, or grouping information indicated by the notes and report them to the Meteorite Nomenclature Committee if the information is valid. We aim to keep the UCLA Cosmochemistry Database dynamically maintained and update information on iron meteorites and recalibrate the compositional data whenever necessary.

## Data Availability

No custom code was used to generate or process the data described in the manuscript.
